# Male principal investigators (almost) don’t publish with women in ecology and zoology

**DOI:** 10.1371/journal.pone.0218598

**Published:** 2019-06-19

**Authors:** Patricia E. Salerno, Mónica Páez-Vacas, Juan M. Guayasamin, Jennifer L. Stynoski

**Affiliations:** 1 Department of Biology, Colorado State University, Fort Collins, Colorado, United States of America; 2 Museo de Zoología, Escuela de Biología, Pontificia Universidad Católica del Ecuador, Quito, Ecuador; 3 Centro de Investigación de la Biodiversidad y Cambio Climático, Facultad de Medio Ambiente, Universidad Tecnológica Indoamérica, Quito, Ecuador; 4 Universidad San Francisco de Quito, Colegio de Ciencias Biológicas y Ambientales, Instituto Biósfera, Laboratorio de Biología Evolutiva, Campus Cumbayá, Quito, Ecuador; 5 Instituto Clodomiro Picado, Universidad de Costa Rica, Coronado, San José, Costa Rica; Universidad de las Palmas de Gran Canaria, SPAIN

## Abstract

Representation of women in science drops substantially at each career stage, from early student to senior investigator. Disparities in opportunities for women to contribute to research metrics, such as distinguished speaker events and authorship, have been reported in many fields in the U.S.A. and Europe. However, whether female representation in scientific contributions differs in other regions, such as Latin America, is not well understood. In this study, in order to determine whether female authorship is influenced by gender or institutional location of the last (senior) author or by subfield within ecology, we gathered author information from 6849 articles in ten ecological and zoological journals that publish research articles either in or out of Latin America. We found that female authorship has risen marginally since 2002 (27 to 31%), and varies among Latin American countries, but not between Latin America and other regions. Last author gender predicted female co-authorship across all journals and regions, as research groups led by women published with over 60% female co-authors whereas those led by men published with less than 20% female co-authors. Our findings suggest that implicit biases and stereotype threats that women face in male-led laboratories could be sources of female withdrawal and leaky pipelines in ecology and zoology. Accordingly, we encourage every PI to self-evaluate their lifetime percentage of female co-authors. Female role models and cultural shifts–especially by male senior authors–are crucial for female retention and unbiased participation in science.

## Introduction

It is well documented that women are underrepresented in science fields, particularly at senior levels [1, 2], and in spite of recent reduction of pay gaps and other advances in gender equity [3–5]. Many studies have investigated how biases generate the phenomenon known as the “leaky pipeline” [3], whereby minorities including women are more likely to be diverted out through the joints between pipes, dropping in representation at every stage in the career pipeline [1, 2]. Female retention is a problem in all STEM (Science, Technology, Engineering and Mathematics) fields, including the biological sciences, where females are overrepresented at the undergraduate level, but withdraw at shockingly high rates at higher career levels [6–9]. Gender underrepresentation is an important issue not only because of social equity and justice [2] but also scientific advancement [10, 11]. However, we still have little understanding of the reasons why women so frequently withdraw from STEM fields and publish less than their male colleagues [4, 12].

In order to properly address female retention in STEM fields and enforce commitments to diversity and inclusion, a closer examination is needed [9]. Disparities in representation and leaky pipelines can be driven by both internal and external sources of bias [1, 2, 13–15]. Stereotype threats, in which individuals doubt their own ability to perform well in a field due to social information, are important internal drivers [16–19]. Unconscious biases against minorities and women are important external drivers. For example, male and female undergraduates consistently rate female peers and instructors lower in academic performance and teaching ability [20, 21]. Also, male and female professors are biased against female students; when hiring future laboratory staff, they ranked application documents with the name “John” about 25% higher than identical documents with the name “Jennifer” [15]. Moreover, female academics are more likely to have their work attributed to a male colleague [22], less likely to have their work cited when in dominant authorship positions [12], and more likely to encounter harassment and discrimination at different stages of their careers [23–25].

Underrepresentation of women is less pronounced in biology than in other STEM fields [5, 26–28], but the leaky pipeline remains an important concern in STEM fields that are female-dominated at the graduate student level [2, 7]. For example, in ecology, a female-dominated field [7], bias against females has been found at the levels of ecological textbooks [6], distinguished speaker invitations [7], and authorship contributions [27]. Authorship order is meaningful in the life sciences, particularly in collaboration-intensive and expensive fields like ecology [8, 29–31]. Despite some regional variation in norms of authorship designation [32–34], the last author usually connotes the head of the lab, the first author connotes the researcher that conducted most of the writing or experiments, and “middle” authors vary in their degree of contribution [8, 30, 33, 35]. Thus, name order influences the credit assigned to researchers, and can point out power imbalances [5, 30, 32].

Gender parity in science becomes even more complex when taking into account cultural and socio-economic differences at the global scale. Previous research has found country and regional indicators of development and gender equality to be poor predictors of female participation in science [36]. Because most studies of academic gender disparity have been conducted in North America and Europe, extrapolation to Latin American ecologists is not straightforward. One might predict gender disparity to be higher in Latin America, as women are relatively less represented in high profile positions such as public office [37, 38]. For example, in Brazil, funding awards and senior-level academic positions in the life sciences are strongly imbalanced against women [39]. Furthermore, because female authorship is higher in countries and regions with lower scientific output [12], career prestige and female representation may be negatively correlated [40]. However, one recent study found the proportion of female authors to be slightly higher among Latin American ecologists as compared to other regions [34].

In this study, we aimed to evaluate gender disparity in the fields of ecology and zoology and to understand variation in gender representation both among subfields and regionally within and outside Latin America. We gathered authorship information from ten peer-reviewed journals in 2002–2016, sampling them in a paired design such that each subfield was represented by journals that focus on publishing research either within or outside Latin America (e.g. Ecology and Biotropica). Based on recent publications, we predicted that women would be underrepresented as authors regardless of region, and that representation would increase over time. We also predicted lower female representation in Latin America. Finally, we predicted that the proportion of female authors would vary by country within Latin America and by gender of last author, but would remain similar across subfields.

## Methods

We obtained information about the authors of articles from ten journals (Table 1), two in general ecology: (1) *Biotropica* (ISSN 0006-3606) and (2) *Ecology* (ISSN 0012-9658); and eight in taxon-specific journals within three zoological subfields, mammalogy: (3) *Acta Zoológica Mexicana* (ISSN 0065-1737) and (4) *Journal of Mammalogy* (ISSN 0022-2372), ornithology: (5) *Ornitología Neotropical* (ISSN 1075–4377) and (6) *The Auk* (0004–8038), and herpetology: (7) *Herpetologica* (ISSN 0018–0831); (8) *Journal of Herpetology* (ISSN 0022-1511); (9) *Phyllomedusa* (ISSN 1519–1397); (10) *South American Journal of Herpetology* (ISSN 1808–9798). We selected journals such that each subfield was represented by at least one journal focused on research in Latin America and one focused on research outside of Latin America (or without a regional focus; Table 1).

**Table 1:** Journals sampled in this study, ordered by impact factor. Table includes information about the field of study, the country of the publisher, the society and publisher of the journal, its impact factor, genders of the chief editorial board (M = male, F = female), and the proportion of female associate editors.

Journal	Field	Country of Publisher	Society/ Publisher	Impact Factor	Chief Editor(s)	Prop. Female Editors
*Ecology*	General Ecology	USA	Ecological Society of America/Wiley	4.62	1 M	0.29 (38/129)
*Biotropica*	General Ecology	Brazil	ATBC/Wiley	2.38	1 M	0.39 (23/59)
*Journal of Mammalogy*	Mammalogy	USA	American Society of Mammalogists/ Oxford Academic	1.94	2 M	0.26 (8/31)
*The Auk*	Ornithology	USA	American Ornithological Society	1.93	2 M, 1 F	0.35 (21/60)
*South American Journal of Herpetology*	Herpetology	Brazil	Sociedad Brasileira de Herpetologia	1.26	3 M	0.17 (9/52)
*Herpetologica*	Herpetology	USA	Herpetologist League/BioOne	1.01	1 M	0.26 (5/19)
*Journal of Herpetology*	Herpetology	USA	SSAR	0.87	1 M, 1 F	0.17 (6/36)
*Phyllomedusa*	Herpetology	Brazil	Universidade de São Paulo	0.35	1 M, 1 F	0.21 (11/52)
*Ornitologia Neotropical*	Ornithology	USA	Neotropical Ornithological Society	0.27	1 M	0.23 (7/31)
*Acta Zoológica Mexicana*	Mammalogy	Mexico	INECOL	0.18	1 M	0.08 (3/40)

We collected author data directly from the title page of 6849 articles published in 2002–2016. Journals varied in the number of volumes and articles printed annually, so we reduced uneven representation by sampling 60 articles among the volumes in a given year in a given journal. For example, in a journal with 6 volumes per year, we used data from the first 10 articles in each of the 6 volumes. In cases in which less than 60 articles were printed in a given year, we included all articles from that year. For each article, we recorded: (1) year; (2) title; (3) number of authors; (4) proportion of female authors; (5) gender of first author; (6) gender of last author; (7) proportion of authors with a Latin American institutional affiliation; and (8) location of last author’s institutional affiliation (outside Latin America or the country in Latin America). We emphasized affiliation of authors rather than ethnic origin to maximize sampling of information available within articles. We excluded articles in which an author's gender was not obvious based on the first name itself or after searching for an academic website with a photo of the author.

Because generalized linear models (GLMs) with binomial error distribution (appropriate for proportion data) were overdispersed, we analyzed data with GLMs using quasibinomial error distribution. To understand author demographics, we determined whether the proportion of female authors, first author gender, last author gender, or proportion of authors based in Latin America as response variables were explained by journal or year as predictor variables. Then, we tested if the gender of the last author, who is generally the head of the research group [8, 30, 33, 35], influenced the proportion of female authors or the gender of the first author, and whether any such effect differed among biological subfields. We also tested whether institutional affiliation of the last author in or out of Latin America influenced the proportion of female authors or the gender of the first or last author. We tested whether last author gender, first author gender, institutional affiliation, or their interaction influenced the number of co-authors. And, we visualized the proportion of female authors by Latin American country. We repeated analyses of the effect of last author gender and institutional affiliation after excluding articles by a single author. We also repeated analyses allowing relationships to vary with journal as a random variable in generalized linear mixed models. Analyses were conducted in R (version 3.5.1 [41]; data and analyses available in S1 Table and S1 Script).

## Results

**Fig 1:**
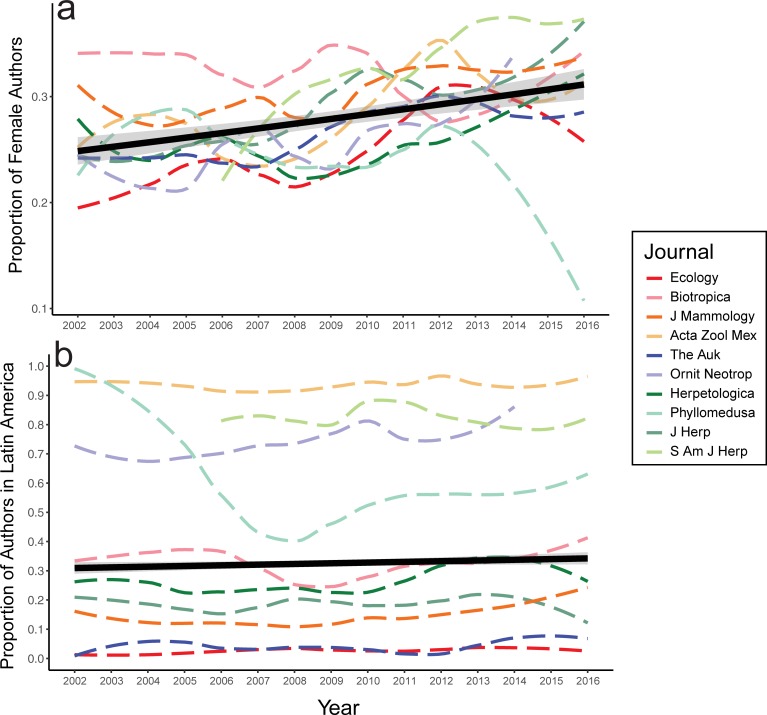
**Change over time by journal in (A) proportion of female authors and (B) proportion of last authors with an institutional affiliation in Latin America.** Gray areas represent 95% confidence intervals.

Women represented 27.9% of authors overall, increasing slightly between 2002 (26.9%) and 2016 (31.4%), with significant increase in the journal *Ecology* and significant decrease in the journal *Phyllomedusa* (Fig 1A; Table 2). Women were first author in 32.5% of articles, increasing from 30.0% in 2002 to 35.2% in 2016, with significant increases in *Ecology* and *Ornitología Neotropical*, and decrease in *Phyllomedusa* (S2 Table). Women were last author in 22.8% of articles, with a statistically non-significant increase from 24.3% in 2002 to 27.9% in 2016 that varied by journal (S3 Table). Institutional affiliation in Latin America did not change across years (32.6% overall, 31.0% in 2002, 28.4% in 2016), but differed significantly among journals (Fig 1B; S4 Table).

**Table 2:** Results of quasibinomial GLM describing the proportion of female authors in ecological journals from 2002–2016.

Coefficient	Estimate	Standard Error	t-value	p-value
(Intercept)	- 42.874	8.610	- 4.979	<0.0001
Year	0.021	0.004	4.875	<0.0001
Biotropica	0.158	0.082	1.919	0.555
Ecology	0.200	0.084	-2.380	0.017
Herpetologica	0.138	0.089	-1.543	0.123
Journal of Herpetology	0.006	0.083	0.077	0.938
Journal of Mammalogy	0.084	0.082	1.020	0.308
Ornitología Neotropical	0.138	0.087	-1.585	0.113
Phyllomedusa	-0.307	0.121	-2.526	0.012
South American J. Herpetology	0.132	0.107	1.232	0.218
The Auk	0.115	0.085	-1.365	0.172

**Fig 2:**
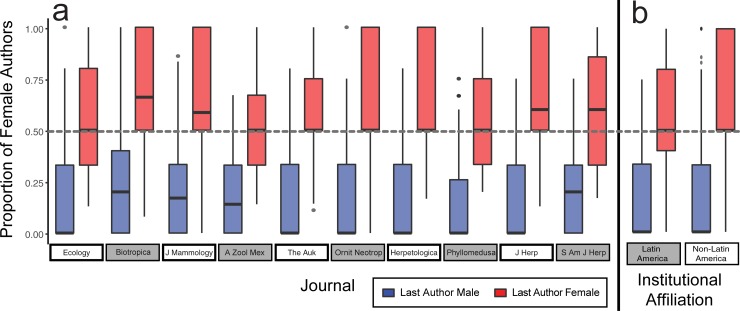
Proportion of female authors in ecological and zoological journals based on gender of last author. Male last authored articles appear in blue and female last authored articles in red. Panels separate data by (A) journal or (B) region of institutional affiliation. Boxplots show median, first and third quartiles, minimum, and maximum.

Last author gender had a strong effect on the proportion of female authors (estimate = 2.07, std. error = 0.036, *t* = 57.31, *P*<0.0001), such that on average 62.8% of authors were female when the last author was female and 17.6% were female when last author was male. The effect of last author gender varied minimally across journals and regional affiliations (Fig 2). Last author gender also predicted first author gender (estimate = 0.779, std. error = 0.059, *t* = 13.12, *P*<0.0001), with female first authors in 28.4% of articles with male last authors and 46.3% in articles with female last authors. First or last author gender and region of institutional affiliation or their interaction did not affect the number of co-authors of an article (all p>0.05; S2 Fig). The proportion of female authors and the effect of last author gender on the proportion of female authors did not differ among biological subfields (all p>0.05; S3 Fig).

**Fig 3:**
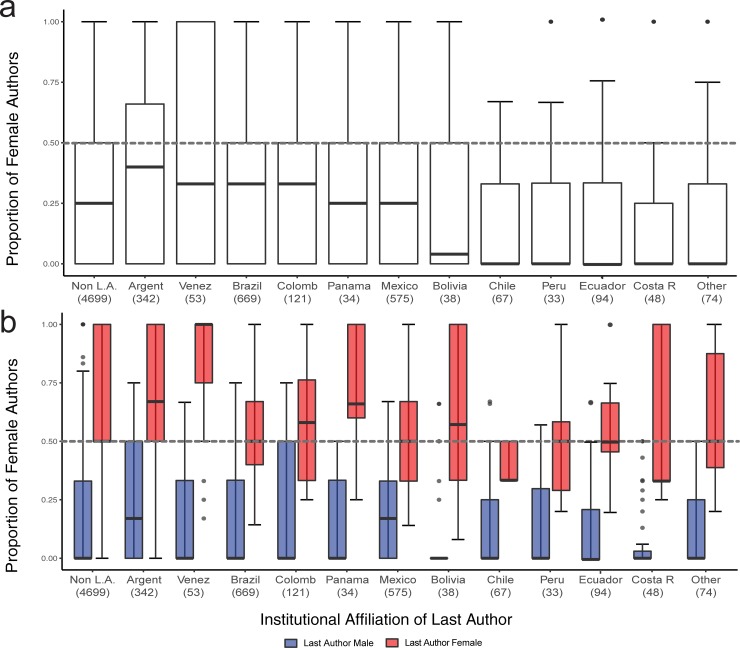
Female authorship in articles that have last authors based in different Latin American countries. (A) Overall proportion of female authors, and (B) proportion of female authors based on gender of last author. Numbers in parentheses indicate the number of articles in this study with last-author affiliation in each country. Countries with fewer than 30 publications (Belize, Cuba, Dominican Republic, El Salvador, Guatemala, Nicaragua, Paraguay, Puerto Rico, Uruguay) are grouped in the category “Other”. Boxplots show median, first and third quartiles, minimum, and maximum.

Last author’s institutional affiliation in or out of Latin America influenced the proportion of female authors (estimate = 0.142, std. error = 0.038, *t* = 3.729, *P* = 0.0002), such that 29.9% of authors were female when the last author was based in Latin America and 27.0% were female when based out of Latin America. Similarly, institutional affiliation influenced first author gender (34.0% in Latin America vs. 31.8% out of Latin America; estimate = 0.11, std. error = 0.055, *t* = 1.89, *P* = 0.05). Institutional affiliation also influenced last author gender (estimate = 0.25, std. error = 0.061, *t* = 4.196, *P*<0.001) such that more articles had female last authors in Latin America (26.0%) than out of Latin America (21.4%). The proportion of female authors varied widely among Latin American countries (Fig 3). Exclusion of 802 single-authored articles (21.3% female authors) and including journal as a random variable in mixed models did not significantly change any of the findings reported here (see S1 Script and S1 Table).

## Discussion

This study identifies an underrepresentation of female authors among peer-reviewed ecological and zoological journals since 2002 (Fig 1), regardless of subfield (Fig 2A), region (Fig 2B), or country in Latin America (Fig 3B). Our findings concur with previous reports showing that females are underrepresented in STEM, including in fields thought to be gender-balanced or female-biased [4, 5, 34]. Gender disparity may be partially attributed to implicit biases that award higher credit for men than women in exchange for equal levels of scientific contribution, as female academics are consistently held to higher standards than male counterparts [27, 42, 43, 44]. Disparity could also be caused by harsher self-assessment resulting from stereotype threats, an important factor in early departure of minorities from STEM [19].

The proportion of female authors has not changed dramatically since 2002 (Fig 1A). While some journals show evidence of improvements in gender parity, we also see evidence of regression, such as in the journal *Phyllomedusa*, where female representation has dropped to almost zero since 2013 (Fig 1A). We observe a significant increase in female first authors over time in only two journals (S2 Table), and no increase in female last authors (S1 Fig). Even though the life sciences are generally female-dominated at the student level [5, 26–28], women are sorely underrepresented in senior and prestigious academic positions [6, 7, 27]. Despite reports of increases in female authorship in biological [4] and medical sciences [25, 36], our findings coincide with other recent studies in biological sciences and ecology [6, 7, 34], and suggest a troubling future for young women in the field.

### Last author gender predicts proportion of female authors

Our most notable finding is that female authorship in ecological journals is strongly dependent on the gender of the last author, as women represent 18% of authors in articles with a male last author and 63% of authors in articles with a female last author. The last author is typically the leader of the research group who oversees the laboratory environment [8, 30, 33, 35], especially in collaboration-intensive STEM fields, where career rank is more important than age in predicting authorship order [30]. Thus, the bias evidenced by our findings suggests that gender-specific and implicit prejudices, harassment, discrimination, and/or stereotype threats may transpire in mentor-mentee relationships [23–25].

Strong bias in female authorship depended on gender of last author throughout our sampling, regardless of zoological subfield (Fig 2A), region (Fig 2B), country (Fig 3B), or journal impact factor (Table 1). The same bias was identified in other recent studies (biological sciences [5]; ecology [34]). However, findings in previous studies either showed less pronounced gender disparity (first female author with 36.2% of male last authored papers and 47.8% with female last authored papers [5]), or were interpreted as a consequence of gender preferences and differences among ecological subfields [34]. We did not find differences in the effect of last author gender on female authorship among subfields (S3 Fig), suggesting that this bias is indeed a widespread issue in ecology and zoology. Gender representation trends were similar among journals with different impact factors and proportions of female editors (except for *Phyllomedusa*; Fig 1A, Table 1). Therefore, we suggest that mentor-mentee relationships, rather than subfield preferences of female authors [34], are important in female withdrawal from this field that has a majority of female students [7].

Interaction with female role models is crucial for female retention in STEM [45, 46]. Although women perceive men as important role models, mentors, and sponsors more frequently than they do women in their careers [47], such data likely reflect sampling bias due to insufficient female role models [48]. Female Ph.D. students in STEM submit fewer articles for publication (3.7) than male PhD students (5.9), but because male students are much more likely than female students to report that faculty motivated them to publish their work (Cohen’s *d* = 0.37), such disparities in publication rate likely point to ineffective mentoring for women [48].

### Scant cross-cultural collaborations and country-level differences suggest complex socioeconomic factors impact gender parity

Authors based in Latin America mostly publish with regional co-authors in Latin American based journals (Fig 1B). Publication of articles by last authors based in Latin America has remained stable over time, except in *Phyllomedusa* where interregional collaborations have increased in recent years. Such regionally limited publication patterns may reduce the impact of Latin American research. For example, only 2% of articles in the high-impact journal *Ecology* have a last author based in Latin America. As editorial boards of ecological journals underrepresent developing countries [49], it is disconcerting to see unwavering regional segregation of collaborations.

Regarding women in ecology in Latin America, we observed slightly more female last authors based in Latin America (26.7%) than out (20.5%), and more female first authors publishing with last authors based in Latin America (33.7%) than out (30.4%). These findings concur with a recent study that examined patterns of gender parity at a larger geographic scale [34]. Studies have found higher female authorship in countries with lower scientific output [12], as well as in countries with an intermediate level of development [36]. Thus, our findings are consistent with the hypothesis that higher female authorship in some countries reflects lower prestige of an academic career, as evidenced by global rankings of universities and research funding [50].

We found considerable differences in female authorship among Latin American countries (Fig 3A), which varied from a median of 0% (Ecuador, Chile, Costa Rica, Peru) to over 30% (Venezuela, Argentina, Brazil, Colombia). We also found differences among countries in the effect of last author gender on the proportion of female authors (Fig 3B). The myriad of political, historical, and societal differences among these countries makes it difficult to speculate about underlying causes behind these authorship patterns. For example, considering public funding, four countries with the most spending on higher education (Brazil, Argentina, Mexico, Chile [50]) varied from over 30% (Argentina, Brazil) to about 25% (Mexico) to near 0% (Chile) female authorship. A recent study also found human development and gender inequality to be poor predictors of gender parity in scientific publications [36]. Considering the potential effect of prestige on female representation, some countries with very low female authorship (Ecuador, Peru, Costa Rica; Fig 3) do not have highly ranked universities [50]. Brazil, the only Latin American country with a university ranked in the top 150 globally [50], had relatively high female authorship (Fig 3). But Chile, which boasts the highest regional citation rate and is a regionally high-ranking university [50], had very low female authorship (Fig 3). Thus, we cannot say with certainty that academic prestige explains all variation in female authorship within Latin America.

At the regional level, we observed that median female authorship is 50% when the last author is female and 0% when the last author is male, regardless of whether the last author is affiliated with an institution inside or outside of Latin America (Fig 2B). Interestingly, at the country level, those countries with the highest female authorship (Fig 3A) also show stronger bias by female lead authors, who publish with a higher proportion of female authors (Argentina 67%, Venezuela 100%; Fig 3B). On the other hand, countries such as Bolivia, Chile, Peru, and Costa Rica that show abysmally low female authorship (Fig 3A) also show very strong male lead-author bias (Fig 3B), suggesting that overall, few females contribute to ecological research in those countries. Thus, higher female authorship in some Latin American countries may be driven by higher gender segregation (i.e. female PIs in ecology publish more with female peers) rather than true gender parity. Similar gender segregation patterns have been reported on editorial boards of ecological journals, where female editors review female-led publications at much higher rates than their male peers [51].

### Conclusions and recommendations

This study makes observations about female authorship since 2002, and offers strong evidence that last author gender, regardless of region of institutional affiliation, predicts whether the other co-authors are likely to be gender-balanced or not. Based on our results, male Principal Investigators (PIs; leaders of research groups) in ecology and zoology appear to carry particular responsibility in reduced opportunities for scientific contribution by women. Below, we provide a few general recommendations in the interest of improving collaborations and mentorship for female students.

First, in ecology, a field overrepresented by female students and underrepresented by women at higher academic levels, female lead researchers are crucial for female retention [2, 5–7]. Female faculty must be actively recruited and incorporated into biology departments and senior positions. Given clear and peer-reviewed evidence that mixed-gender teams generate more effective, innovative, and impactful research [10, 11], such recruitment is also crucial for addressing growing ecological-societal problems around the world.

Second, given that male faculty are more reluctant to accept published data on gender biases and discrimination than female faculty [52], female faculty need to work actively to keep young female ecologists in this field. Stereotype threats–impacts of perceived judgment based on one’s group identity rather than on their personal merit–are better predictors of early departure from STEM than lack of academic preparation or true underperformance [19]. Stereotype threats can raise levels of test anxiety, and produce gender disparities in biology exam performance [53]. Helping young female scientists to understand, anticipate, and accept those inherent internal threats and sources of anxiety can improve retention by making it clear that such psychosocial threats are shared experiences [54]. Also, active learning pedagogies [55] and undergraduate research experiences [56] greatly increase self-efficacy and thus reduce stereotype threats. Finally, assortative gender patterns, in which female PIs deliberately recruit female students and junior scholars, may be necessary to improve inclusion and retention of women and other minority groups [9, 51].

Third, academic institutions should monitor the relationships and mental well-being of women in labs with male PIs to assess whether measures should be taken to reduce harassment and discrimination [24], and should also offer resources for counseling and managing impostor syndrome. Academics suffer from exceptionally high rates of mental illness [57], so facilitating and reducing stigma surrounding counseling resources for both men and women would have the added benefit of reducing female attrition.

In closing, we would like to re-highlight the finding that low female authorship in ecological and zoological publications is directly related to the gender of PIs, regardless of subfield or regional affiliation. Articles with female last authors had almost four times as many female co-authors as articles with male last authors. Such systematic exclusion of women from papers with male last authors suggests a need for male PIs to take a more active role in self-assessment, and to actively seek resources and training about disparities and implicit biases their own students may face. We encourage every PI to take a moment to calculate their own lifetime proportion of female co-authors and decide if they are satisfied with the result. Changes made by PIs and institutions to ameliorate the problem should aim to reduce disparities in motivation, participation, opportunities, and credit for research papers, funding, and conferences among male and female mentees and collaborators.

## Supporting information

S1 Table Raw data about authorship of articles from journals in this study. (CSV)

S2 Table Results of quasibinomial GLM describing women as first authors in ecological journals from 2002–2016. (XLSX)

S3 Table Results of quasibinomial GLM describing women as last authors in ecological journals from 2002–2016. (XLSX)

S4 Table Results of quasibinomial GLM describing authors based at Latin American institutions in ecological journals from 2002–2016. (XLSX)

S1 Script R code used to analyze data in this study. (R)

S1 Spanish Abstract (DOCX)

S1 Fig Representation of female last authors in 2002–2016 by journal. (PDF)

S2 Fig Number of authors in a given article by institutional affiliation and gender of last author. (PDF)

S3 Fig Proportion of female authors by subfield. (PDF)
