# Correction: Male principal investigators (almost) don’t publish with women in ecology and zoology

**DOI:** 10.1371/journal.pone.0233803

**Published:** 2020-05-21

**Authors:** Patricia E. Salerno, Mónica Páez-Vacas, Juan M. Guayasamin, Jennifer L. Stynoski

After publication of this article [1], concerns were raised regarding the methodology and statistical analyses, in particular about (1) inclusion of single author papers in analysis, (2) inclusion of last author in the category of “proportion of female co-authors”, (3) empirically unrealistic null expectation and (4) improperly curated data. In addition, a post-publication statistical review revealed concerns with the statistical analysis. Additional analyses were conducted to address these issues, which were found to impact some of the reported results but which did not alter the validity of the overall conclusions. Here, the authors provide additional information to clarify these issues.

The original analysis identified a significant effect of time on the proportion of female authors (see Fig 1A, Table 2, and the first sentence of the Results), such that the proportion of female authors increased from 26.9% to 31.4% between 2002 and 2016. However, the original analysis did not consider the effect of the interaction between the year of publication and last author gender. In an additional quasibinomial model with 802 single-authored papers omitted, last author gender (estimate = 49.7, p = 0.003), year (estimate = 0.03, p < 0.001), and their interaction (estimate = -0.02, p = 0.004) all affected the proportion of female authors. Including single-authored papers in the analysis did not alter these conclusions. Thus, the addition of the interaction of the effect of time changes the estimated parameters, but does not alter the conclusions about the significant and substantial effect of last author gender on the proportion of female authors.

**Table 2:** Results of quasibinomial GLM describing the proportion of female authors in ecological journals from 2002–2016.

Coefficient	Estimate	Standard Error	t-value	p-value
(Intercept)	-42.874	8.610	- 4.979	<0.0001
Year	0.021	0.004	4.875	<0.0001
Biotropica	0.158	0.082	1.919	0.055
Ecology	-0.200	0.084	-2.380	0.017
Herpetologica	-0.138	0.089	-1.543	0.123
Journal of Herpetology	0.006	0.083	0.077	0.938
Journal of Mammalogy	0.084	0.082	1.020	0.308
Ornitología Neotropical	-0.138	0.087	-1.585	0.113
Phyllomedusa	-0.307	0.121	-2.526	0.012
South American J. Herpetology	0.132	0.107	1.232	0.218
The Auk	-0.115	0.085	-1.365	0.172

The original analysis determined that the number of co-authors was not affected by first or last author gender or region of institutional affiliation or their interaction (see the fourth sentence of the second paragraph of the Results). However, it did not directly consider whether the number of co-authors influenced the effect of last author gender on the proportion of female authors. To evaluate this effect, the authors used the same quasibinomial model with the interaction effect as indicated above, but assigned a weight to each data point based on the number of co-authors in the article. In this model, last author gender (estimate = 82.2, p < 0.001), year (estimate = 0.04, p < 0.001), and their interaction (estimate = -0.04, p < 0.001) all affected the proportion of female authors. Therefore, although the model estimates of the effect of last author gender differ among the three models (original analysis, 109.7; interaction with year included, 49.7; weighted by number of authors, 82.2), all of these analyses indicate a significant effect of last author gender on the proportion of female authors, and a substantial effect size.

There are typographical errors in Table 2. Some negative signs are missing from the estimate values for the journals *Ecology*, *Herpetologica*, *Ornitología Neotropical*, and *The Auk*. Please see the correct Table 2 here.

As a result of these corrections, there are errors in the text associated with Table 2 and S2 Table. The following sentences need to be amended:

- First sentence of the Results section: Women represented 27.9% of authors overall, increasing slightly between 2002 (26.9%) and 2016 (31.4%), with significantly lower female authorship in *Ecology and Phyllomedusa* than in *Acta Zoológica Mexicana* (Fig 1A; Table 2).
- Second sentence of the Results section: Women were first author in 32.5% of articles, increasing from 30.0% in 2002 to 35.2% in 2016, with significantly lower female first authorship in *Ecology*, *Ornitología Neotropical*, and *Phyllomedusa* than in *Acta Zoológica Mexicana* (S2 Table).
- Third sentence of the second paragraph of the Discussion section: We observe that some journals such as *Ecology* and *Phyllomedusa* maintain lower female authorship relative to others such as *Acta Zoológica Mexicana* (S2 Table), and did not observe an increase in female last authors (S1 Fig).

Four of 6849 data points (0.00058% of papers) were miscoded in the dataset and can be seen in the original Fig 2 as points where Last Author Male articles appear as having 100% female authors. The results are unaffected by the correction of these errors in the dataset.

For the sake of clarity, the authors highlight that the removal of 802 single authored papers alters the estimated coefficients of statistical models with a somewhat reduced effect size concerning the effect of last author gender on the proportion of female authors; however, this does not affect the statistical significance or conclusions of the study (see Line 170 in original R script and the last sentence of the Results). Focusing on raw data values rather than model parameters, the omission of single-authored papers shifts the percentage of female authorship on papers with male last authors from 17.6% to 19.9%. Therefore, the percentages both with and without single-authored papers are consistent with the estimation of “less than 20%” stated in the Abstract as a key finding of this study.

The authors clarify that the null expectation of the study was that the proportion of female authors would be similar among male- and female-last authored papers, and not that the proportion of female authors would be 50%.

Lastly, the authors restate from the original Methods that this study’s analysis focused on the proportion of all authors of each article as a data point representative of the gender balance of the entire research team, and not on the proportion of female authors of the article besides the last author.

## References

[1] Salerno PE, Páez-Vacas M, Guayasamin JM, Stynoski JL (2019) Male principal investigators (almost) don’t publish with women in ecology and zoology. PLoS ONE 14(6): e0218598 10.1371/journal.pone.0218598 31216351 PMC6583967

