# Supplementary material for: Male principal investigators (almost) don’t publish with women in ecology and zoology
Source: PLoS One. 2019 Jun 19;14(6):e0218598. doi: 10.1371/journal.pone.0218598 (PMC6583967; doi:10.1371/journal.pone.0218598)
Supplement: S1 Spanish Abstract — (DOCX) [file pone.0218598.s006.docx]

**RESUMEN**

La representación de mujeres en la ciencia se ve reducida sustancialmente en cada etapa de la carrera, desde etapas tempranas como estudiantes de pregrado hasta llegar a posiciones como jefas de laboratorios. En Estados Unidos y Europa se han reportado disparidades en las oportunidades disponibles para que las mujeres contribuyan a las métricas de investigación, así como ponencias magistrales y autoría. Sin embargo, no se ha evaluado si la representación femenina en las contribuciones científicas difiere según la región, ejemplo de ello es América Latina. Para entender si existen diferencias en cuanto a autoría femenina de acuerdo a la localidad de la institución y el género del último autor, o de subcampo dentro de ecología, evaluamos la información relacionada con la autoría obtenida de 6849 artículos en diez revistas ecológicas y zoológicas tanto dentro como fuera de Latinoamérica. Encontramos que la autoría femenina ha aumentado marginalmente desde el año 2002 hasta el 2016 (del 27% al 31%), y que varía de un país a otro en América Latina, pero no a nivel regional dentro y fuera de América Latina. Encontramos que el género del último autor es un buen predictor de la participación de mujeres como autores en todas las revistas y regiones, ya que en los grupos de investigación dirigidos por mujeres más del 60% de los coautores son mujeres, mientras que en los grupos dirigidos por hombres apenas el 20% de los coautores son mujeres. Nuestros hallazgos sugieren que los sesgos implícitos y las amenazas relacionadas a los estereotipos que enfrentan las mujeres en los laboratorios dirigidos por hombres son una fuente importante de abandono femenino de la ecología y zoología como carreras. Como tal, recomendamos a los jefes de laboratorio a autoevaluar en porcentaje de colaboradoras femeninas durante su carrera. Resulta crucial para la retención y permanencia de las mujeres en esta disciplina que existan mujeres como modelos a seguir, asi como cambios culturales particularmente por parte de los hombres jefes de laboratorios.
