# Supplementary figures and images for: Male principal investigators (almost) don’t publish with women in ecology and zoology
Source: PLoS One. 2019 Jun 19;14(6):e0218598. doi: 10.1371/journal.pone.0218598 (PMC6583967; doi:10.1371/journal.pone.0218598)

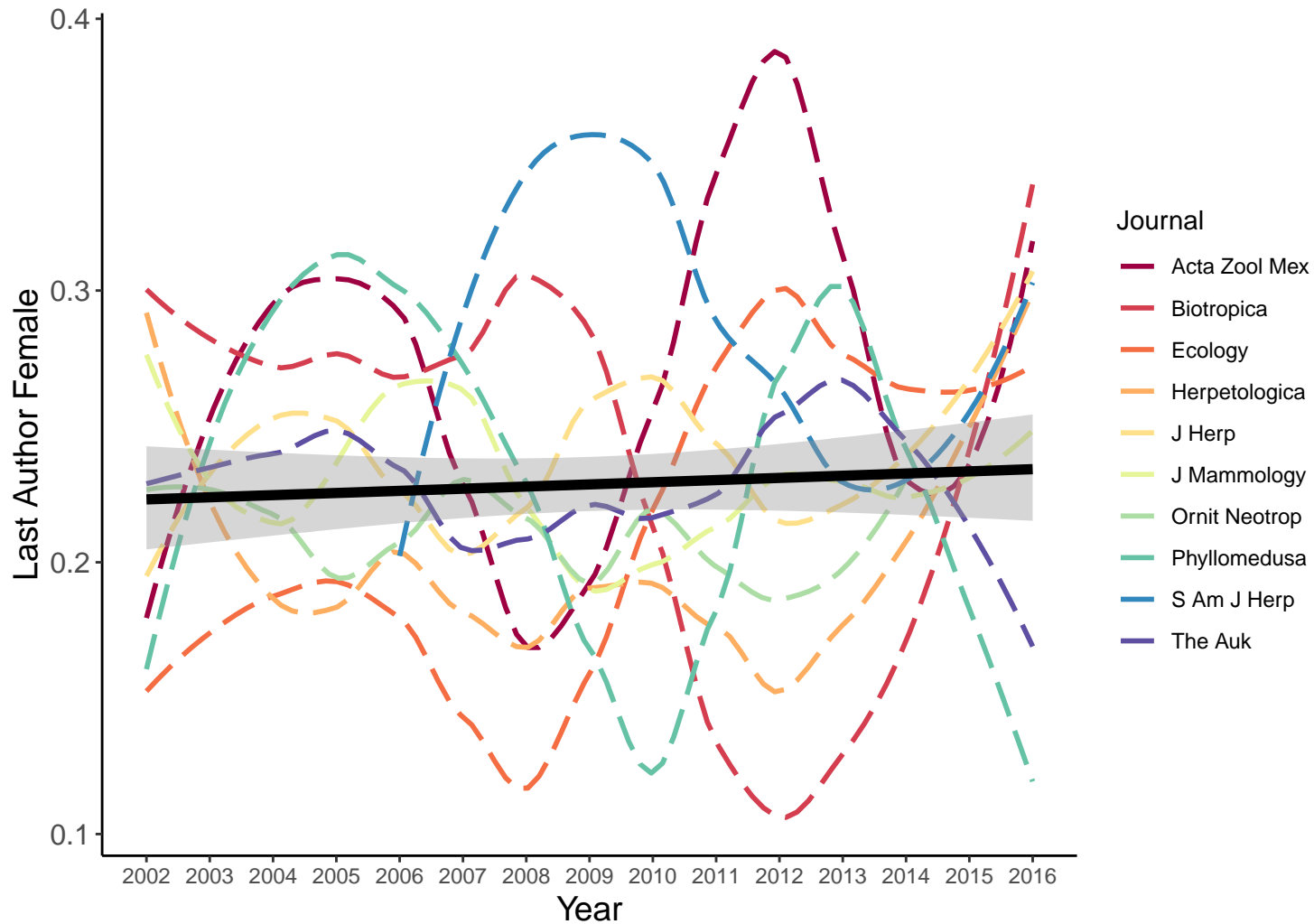

Supplement: S1 Fig — (PDF) [file pone.0218598.s007.pdf]

Number of Authors per Article

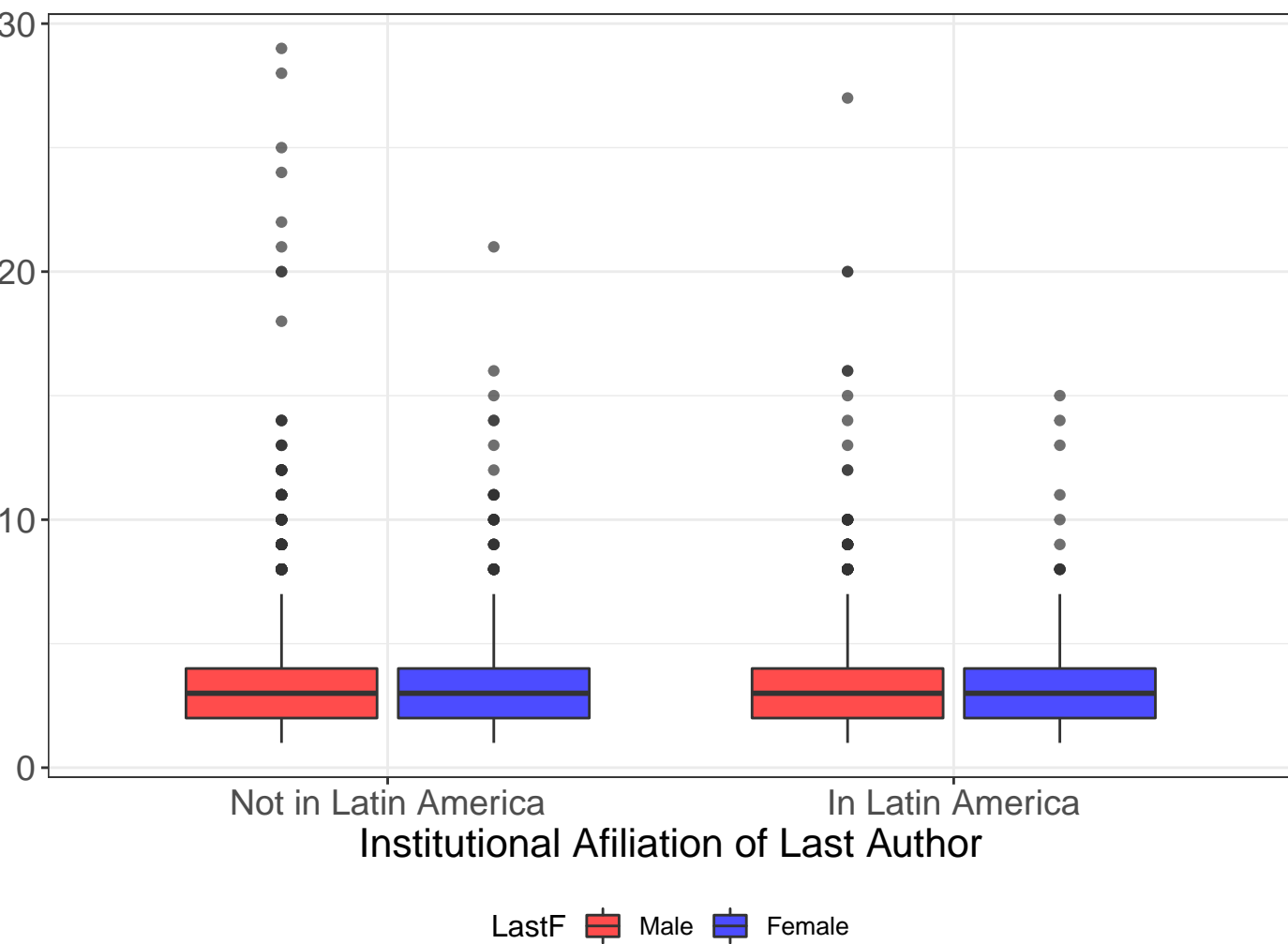

Supplement: S2 Fig — (PDF) [file pone.0218598.s008.pdf]
