# Supplementary material for: Male principal investigators (almost) don’t publish with women in ecology and zoology
Source: PLoS One. 2019 Jun 19;14(6):e0218598. doi: 10.1371/journal.pone.0218598 (PMC6583967; doi:10.1371/journal.pone.0218598)
Supplement: S3 Fig — (PDF) [file pone.0218598.s009.pdf]

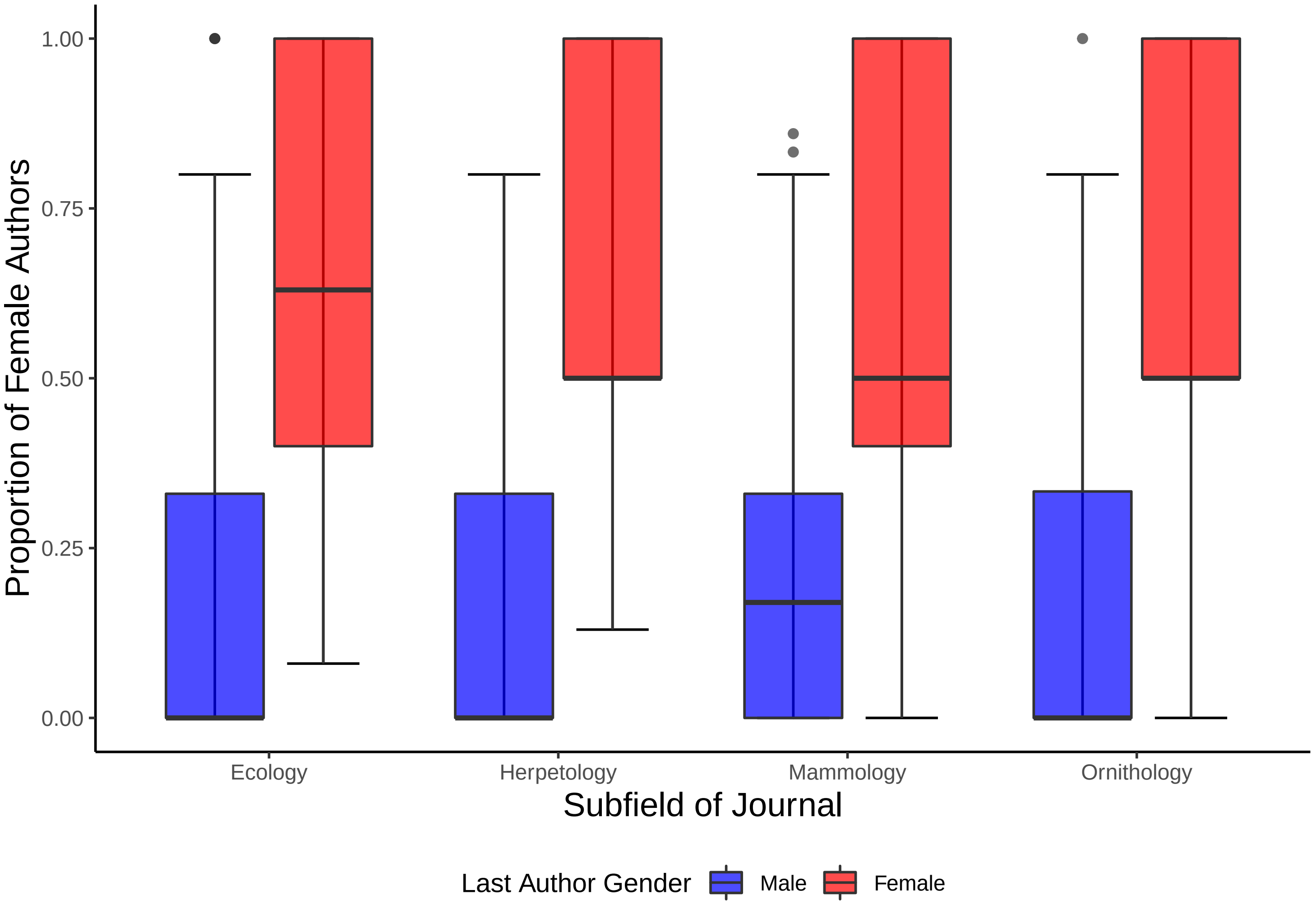

Proportion of Female Authors
1.00
0.75
0.50
0.25
0.00
Ecology
Herpetology
Mammology
Ornithology
Subfield of Journal
Last Author Gender
Male
Female
